# Association of Angiotensin II Type 1 Receptor (A1166C) Gene Polymorphism and Its Increased Expression in Essential Hypertension: A Case-Control Study

**DOI:** 10.1371/journal.pone.0101502

**Published:** 2014-07-03

**Authors:** Sudhir Chandra, Rajiv Narang, Vishnubhatla Sreenivas, Jagriti Bhatia, Daman Saluja, Kamna Srivastava

**Affiliations:** 1 Dr. B R Ambedkar Centre for Biomedical Research, University of Delhi, Delhi, India; 2 Department of Cardiology, All India Institute of Medical Sciences, New Delhi, India; 3 Department of Biostatistics, All India Institute of Medical Sciences, New Delhi, India; 4 Department of Pharmacology, All India Institute of Medical Sciences, New Delhi, India; Sanjay Gandhi Medical Institute, India

## Abstract

**Objectives:**

Hypertension is one of the major cardiovascular diseases. It affects nearly 1.56 billion people worldwide. The present study is about a particular genetic polymorphism (A1166C), gene expression and protein expression of the angiotensin II type I receptor (AT1R) (SNP ID: rs5186) and its association with essential hypertension in a Northern Indian population.

**Methods:**

We analyzed the A1166C polymorphism and expression of AT1R gene in 250 patients with essential hypertension and 250 normal healthy controls.

**Results:**

A significant association was found in the AT1R genotypes (AC+CC) with essential hypertension (χ^2^ = 22.48, p = 0.0001). Individuals with CC genotypes were at 2.4 times higher odds (p = 0.0001) to develop essential hypertension than individuals with AC and AA genotypes. The statistically significant intergenotypic variation in the systolic blood pressure was found higher in the patients with CC (169.4±36.3 mmHg) as compared to that of AA (143.5±28.1 mmHg) and AC (153.9±30.5 mmHg) genotypes (p = 0.0001). We found a significant difference in the average delta-CT value (p = 0.0001) wherein an upregulated gene expression (approximately 16 fold) was observed in case of patients as compared to controls. Furthermore, higher expression of AT1R gene was observed in patients with CC genotype than with AC and AA genotypes. A significant difference (p = 0.0001) in the protein expression of angiotensin II Type 1 receptor was also observed in the plasma of patients (1.49±0.27) as compared to controls (0.80±0.24).

**Conclusion:**

Our findings suggest that C allele of A1166C polymorphism in the angiotensin II type 1 receptor gene is associated with essential hypertension and its upregulation could play an important role in essential hypertension.

## Introduction

Hypertension is the most common risk factor for cardiovascular disease, stroke, and renal disease due to the elevated levels of both systolic and diastolic blood pressure [1]–[4]. It is estimated that by the year 2025 prevalence of hypertension will be increased by 60% as compared to year 2000 and nearly 1.56 billion individuals will have hypertension worldwide [5]. Trends in hypertension prevalence and its epidemiology in India have been critically reviewed earlier [6]–[8]. Hypertension is responsible for 24% of all coronary heart disease deaths and 57% of all the stroke deaths in India [9]. Essential hypertension is considered to result from the interaction of environmental and genetic factors, with approximately 30% of the inter-individual variability in blood pressure being genetically determined [10]–[11]. The renin-angiotensin-aldosterone system (RAAS) plays a significant role in blood pressure regulation, and has been suggested to be involved in essential hypertension [12]. Angiotensin II receptors are of two types: type 1 (AT1) receptor and type 2 (AT2) receptor [13]. Receptor binding studies provide important information regarding the distribution of AT1 and AT2 receptors and the sites of action and physiological roles of angiotensin [14]–[15]. The peptide hormone Angiotensin II is a potent vasoconstrictor that exerts its actions through angiotensin II type 1 receptor (AT1R). Essential hypertension is associated with the genetic mutations in genes of RAAS, such as the AT1R gene [16]–[17]. Polymorphism in RAAS gene such as AT1R A1166C (SNP ID: rs5186) [18] angiotensinogen (AGT) M235T and angiotensin converting enzyme insertion/deletion (ACE I/D) [19]–[21] has been widely studied and found to be associated with essential hypertension. AT1R gene is composed of five exons located on chromosome 3q, where the first four exons encode the 5′-untranslated region (5′- UTR) [22]. A polymorphism in the 3′ untranslated region of AT1R gene leads to the transversion of adenine (A) to cytosine (C) base at the 1166 position [23]–[24]. AT1R gene plays an important role in the cardiovascular system, such as vasoconstriction, smooth muscle cell growth and cellular hypertrophy governed by major signaling mechanisms [25]. AT1R A1166C gene polymorphism is associated with essential hypertension in Caucasions [23]–[24], [26]–[27], Iranian [28] and Asian populations [29]–[31] while other studies have shown no significant association in Nigerian [32], Kazakans [33], Japanese [34] and South Indian Tamilian population [35]. A positive correlation was reported between AT1R gene expression and Systolic blood pressures (SBP) in spontaneously hypertensive rat [36]. In a separate study, an elevated level of AT1R gene expression in the brainstem of spontaneously hypertensive rat was observed [37]. The transcriptional activity and mRNA stability of AT1R gene is regulated by specific RNA-binding proteins [38]. The role of AT1R protein expression in the regulation of blood pressure has been reported in recent studies [39]–[40]. It was reported that arterial blood pressure can be effectively reduced and the vascular activity reversed by viral gene delivery of AT1R antisense in adult spontaneously hypertensive rat [41].

The genetic polymorphism (A1166C) and gene expression of AT1R in patients with essential hypertension has not been reported so far in a Northern Indian population. In the current study we have investigated the association of AT1R genetic polymorphism with essential hypertension. The expression of AT1R at RNA and protein levels in individuals with essential hypertension was also compared with that of normal controls in Northern Indian population. The present study is a step towards investigating the circulating prognostic markers, if any, in essential hypertension.

## Materials and Methods

### Study population

Earlier study in Chinese Han population indicated that the allele A frequency of the AT1R gene is 96.6% among controls and 90.8% among patients with essential hypertension [47]. Using this information, the sample size was calculated using PS-Power and sample size calculation version 3.0 software. Accordingly, 275 cases and 275 controls were required to detect a statistically significant difference in the allele A frequency at two sided 5% α error and with 80% power. In our study, 250 unrelated essential hypertensive patients (males and females, aged 25–60 years) were recruited from the Outpatient clinics of hypertension, Department of Cardiology, All India Institute of Medical Sciences, New Delhi, India. These patients were residents of North India for at least three generations. No subject in this study, case or control, was receiving anti – hypertensive therapy of any sort. Newly diagnosed hypertensive patients with systolic blood pressure (SBP) more than 140 mmHg and/or diastolic blood pressure (DBP) more than 90 mmHg on two or more consecutive visits were considered as hypertensives. Patients with history of diabetes mellitus, hyperlipidaemia, liver or renal disease, congestive cardiac failure and recent episode of myocardial infarction were excluded from the study. Patients with pregnancy and lactation and receiving medications for other indications that could affect blood pressure were also excluded.

The control group consisted of 250 (males and females, aged between 25–60 years) unrelated healthy volunteers. These subjects had no personal or family history of hypertension and other cardiovascular diseases in first-degree relatives and had SBP<140 mmHg and DBP<90 mmHg. Healthy volunteers who visited the Outpatient clinics with minor illness without hypertension, diabetes mellitus, hyperlipidaemia and family history of hypertension in previous records were recruited as controls. None of the subjects in the control group was receiving treatment for heart disease or hormone-replacement therapy during the time of the study. Plasma lipid profile and blood glucose level were measured after overnight fasting in both hypertensives and normotensives to rule out diabetes and hyperlipidaemia. All the participants were interviewed using a questionnaire with regard to their lifestyle, smoking, alcohol consumption, food intake and their family history of hypertension. In all subjects, height was measured to the nearest centimeter and weight to the nearest 0.1 kg which was used for calculation of BMI (kg/m2).

For measurement of blood pressure, mercury sphygmomanometer was used and guidelines given by JNC-VII were followed. Blood pressure (BP) was measured by the same doctor, two minutes apart, three times in the right arm using standard sphygmomanometer with a medium- or a large-size cuff, according to the subject’s arm circumference (cuff bladder encircling at least 80% of the arm) after the subjects rested for 10 min and the average reading was recorded. The diagnosis of hypertension was based on at least two BP measurements per visit and at least two visits so as to exclude false diagnosis due to variability that may be caused by various factors at any one point in time. The distribution of end digit preference for systolic and diastolic blood pressures is given in table S1. The blood samples were collected from the antecubital vein between 8 a.m. and 10 a.m., in a sitting position, after 12 hours of fasting and alcohol absence. The biochemical evaluation was carried out in the same laboratory that followed the criteria of the World Health Organization Lipid Reference Laboratories.

Written informed consent was obtained from all the participants recruited for the study. The study was conducted in accordance with the guidelines of the Helsinki Declaration. An approval of ethics committee of All India Institute of Medical Sciences, New Delhi, India was obtained prior to the study. The outcome of the study was not used to effect the treatment given to the patient.

### Sample collection and processing

Five milliliter (5 ml) of peripheral venous blood was collected from all the participants in an ethylene diamine-tetra acetic acid (EDTA) vial after 12 hours of fasting. One ml of whole blood was used for RNA extraction and remaining volume of blood was used for DNA extraction and separation of plasma by centrifugation at 520×g for 10 min for biochemical analysis and protein expression.

### DNA extraction and Genotyping

Genomic DNA was extracted using the Flexigene DNA kit (QIAGEN) according to the manufacturer’s instructions. AT1R gene was amplified by polymerase chain reaction (PCR) using the following primer pair:

5′-ATAATGTAAGCTCATCCACC- 3′ (forward).

5′-GAGATTGCATTTCTGTCGGT- 3′ (Reverse).

The PCR amplified 359-bp products were analyzed for genotyping by restriction fragment length polymorphism using the restriction enzyme Dde1. Individuals homozygous for allele, i.e AA, a 359- bp band appeared on the gel, homozygous CC (220-bp band and a 139-bp band appeared on the gel) and heterozygous AC (all 3 bands, i.e 359- bp, 220-bp and 139-bp appeared on the gel).

### RNA extraction and cDNA preparation

Total RNA was extracted from whole blood samples by QIAamp RNA Blood Mini Kit (QIAGEN) according to the manufacturer’s instructions and Quantified by using Nanodrop (ND-1000). The yield of total RNA was in the range of 600–800 ng/µl, from which 500 ng of total RNA was used for cDNA synthesis using First Strand cDNA Synthesis kit (Fermentas) according to the manufacturer’s protocol.

### Angiotensin II type 1 receptor (AT1R) gene expression

The AT1R gene expression was determined by semi-quantitative and quantitative real time PCR. Semi-quantitative PCR was performed by using Mastercycler gradient (eppendorf). Levels of AT1R gene expression were quantified by densitometric analysis in terms of Integrated densitometric value (IDV) by imageJ software (NIH). The IDV for AT1R gene in study subjects was normalized to 18S rRNA gene expression. Real time quantitative PCR was performed by using ABI 7300 Real Time PCR System (Applied Biosystems) with SYBR green (Eurogentec) PCR Core reagents. Amplification was performed in triplicates in 15 µl volume, including 1 µl cDNA, 1 pmol of each primer in 2X Mesa green PCR Master mix (Eurogentec). To amplify human AT1R transcript the following primers (Sigma) based on gene bank sequence (NCBI Reference Sequence: NM_000685.4) were used.

Forward 5′- AGGGCAGTAAAGTTTTCGTG -3′.

Reverse 5′- CGGGCATTGTTTTGGCAGTG -3′.

18S rRNA served as a housekeeping gene to access the overall cDNA content and as endogenous control using following primers:

Forward 5′-GTGGTGTTGAGGAAAGCAGACA-3′.

Reverse 5′-TGATCACACGTTCCACCTCATC-3′.

Real time PCR was performed in triplicate for each sample according to the protocol as follows: After an initial holding step of 2 min at 50°C and 10 min at 95°C, samples were cycled 40 times at 95°C for 15 sec and 60°C for 1 min. The melting curve analysis of amplified product was used to confirm the specificity of real time PCR assay. Difference between the CT values (delta-CT value) obtained for the gene of interest and normalizer or housekeeping gene was calculated. The cycle threshold (CT) is defined as index of number of cycles required for the fluorescent signal to cross the threshold whereas delta-CT value is the difference between the CT values obtained for the gene of interest and normalizer or housekeeping gene. The AT1R gene expression was compared in controls and patients group in terms of fold difference determined by delta-delta-CT equation according to the formula:
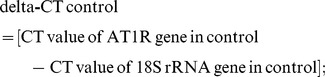


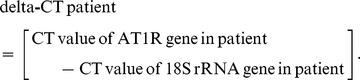


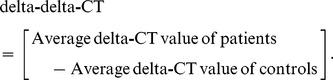


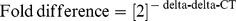



### Isolation of protein and western blot

Total protein was isolated from plasma by acetone precipitation method using Bicinchoninic acid (BCA) protein estimation kit (Banglore Genei, India). Equal amount of proteins (50 µg) were separated on 12% SDS-polyacrylamide gels and electrobloted on to PVDF membranes (MDI, India). The membranes were blocked overnight in 3% Bovine Serum Albumin (BSA), washed with Phosphate buffered saline containing 0.5% Tween-20 (PBST) and incubated with anti hAT1R (1∶500 dilution) primary antibody (sc-1173, Santa Cruz Biotechnology, USA) for 2–3 hours. Thereafter, blot was washed thrice with PBST before incubating with horseradish peroxidase–conjugated secondary antibody (1∶5000 dilutions; sc-2357, Santa Cruz Biotechnology, USA) and detected by chemiluminescence detection (WEST-ZOL Plus, Intron Biotechnology, Korea). Levels of AT1R protein expression were quantified by densitometric analysis by imageJ software (NIH) in terms of integrated densitometric value (IDV). The protein expression was compared by average ratio of IDV of AT1R protein and β-actin in the study subjects.

### Statistical analysis

All statistical analysis was performed using Stata program, version 12.1 and GraphPad Prism version 5 (GraphPad Software Inc., San Diego, CA, USA). Chi-square goodness of fit was used to verify the agreement of observed genotype frequencies with those expected (Hardy–Weinberg equilibrium). The analysis of variance (ANOVA) was used to calculate the difference between genotype groups using Bonferroni’s method for multiple comparisons between genotype classes. An odds ratio at [95% confidence intervals (CI)] was calculated as an index of the association of the gene with the disease. Results for gene and protein expression analysis are expressed as mean±SD. Statistical significance was defined as a p-value <0.05.

## Results

### Baseline characteristics of the study subjects

The patients with essential hypertension (N = 250) and normal healthy controls (N = 250) were recruited in our study. The number of male subjects was higher as compared to the number of female subjects in both the study groups. The lipid profile values such as Total cholesterol (TC), Triglyceride (TG), High Density Lipoprotein cholesterol (HDL) and Low Density Lipoprotein cholesterol (LDL) were comparable in patients and controls. Systolic blood pressures (SBP) in patients were significantly higher (151.0±12.5 mm Hg) than that of controls (120±3.8 mm Hg). Similarly, Diastolic blood pressures (DBP) in patients were higher (94.8±8.8 mm Hg) than in controls (80.4±2.9 mm Hg) **(** **Table 1** **)**.

**Table 1:** Baseline characteristics of the study subjects.

Parameters	Patients (N = 250)	Controls (N = 250)	p
Sex (M/F)	164/86	153/97	0.42
Age (years)	49.2±12.0	52.1±7.0	0.26
BMI, (Kg/m^2^)	18.4±2.9	18.03±3.4	0.88
Heart Rate (Beats/min)	74.7±9.6	72.3±4.9	0.34
Blood glucose (mg/dl)	91.7±16.5	92.4±14.7	0.86
Blood Urea (mg/dl)	20.3±3.9	18.3±3.7	0.14
Serum Creatinine	0.97±0.2	0.95±0.2	0.22
LDL cholesterol (mg/dl)	88.5±20.9	91.8±26.8	0.26
HDL cholesterol (mg/dl)	41.5±6.5	38.8±6.5	0.13
Total cholesterol (mg/dl)	169.8±30.8	170.4±23.8	0.56
Triglyceride (mg/dl)	160±37.0	159±42.0	0.35
Systolic blood pressure (SBP) mm Hg	151.0±12.5	120±3.8	0.0001*
Diastolic blood pressure (DBP) mm Hg	94.8±8.8	80.4±2.9	0.0001*

BMI, Body mass index; HDL, High density lipoprotein; LDL, Low density lipoprotein.

Patients group were compared with controls with t-test of significance or by chi-square test;

*p<0.05 is considered to be significant.

### Distribution of genotype and allele frequencies of AT1R A1166C

The distribution of genotypes in the patient (χ^2^ = 0.10, p>0.05) and control (χ^2^ = 0.092, p>0.05) groups was in accordance with the Hardy-Weinberg equilibrium. The heterozygous genotypic pattern (AC) is more frequent in patients while in controls the most frequent genotype was AA. The observed allele frequencies in patients were 0.63 and 0.37 for A and C alleles respectively, whereas 0.79 and 0.21 was observed in control groups. There was a significant association found in the AT1R genotypes (AC+CC) with essential hypertension [χ^2^ = 22.48, p = 0.0001, Odds ratio = 2.4 (1.65 – 3.50) at 95% CI]. The adjusted odds ratio for A Vs C allele frequencies was found to be 2.09 (1.56–2.80) [χ^2^ = 28.13, p<0.0001 at 95% CI] **(** **Table 2** **)**.

**Table 2:** Genotype and Allele Frequencies of A1166C variant of the Angiotensin II type 1 receptor gene in the study subjects.

Subjects	Genotypes, n(%)				Adjusted odds ratio at 95% Confidence Intervals for genotypes:
	AA (%)		AC (%)	CC (%)	
Controls (n = 250)	155 (62)		83 (33.2)	12 (4.8)	AA Vs AC = 2.1[1.4–3.0] p<0.0001 AA Vs CC = 4.4[2.2–9.0] p<0.0001 AA Vs AC+CC = 2.4[1.65–3.50] p<0.0001
Patients (n = 250)	101 (40.4)		114 (45.6)	35 (14)	
**Subjects**	**Allele frequency**				**Adjusted odds ratio at 95% Confidence Intervals for alleles:**
	**A**	**C**			
Controls	0.79	0.21			A Vs C = 2.09 [1.56–2.80] p<0.0001
Patients	0.63	0.37			

Patients groups were compared with controls with chi-square (χ^2^) test at one degree of freedom with Odds ratio adjusted for age and sex in both genotypes and alleles. p<0.05 is considered to be significant.

### Intergenotypic (A1166C variant of the Angiotensin II type 1 receptor gene) variations in systolic and diastolic blood pressure in patients with essential hypertension

**Figure 1:**
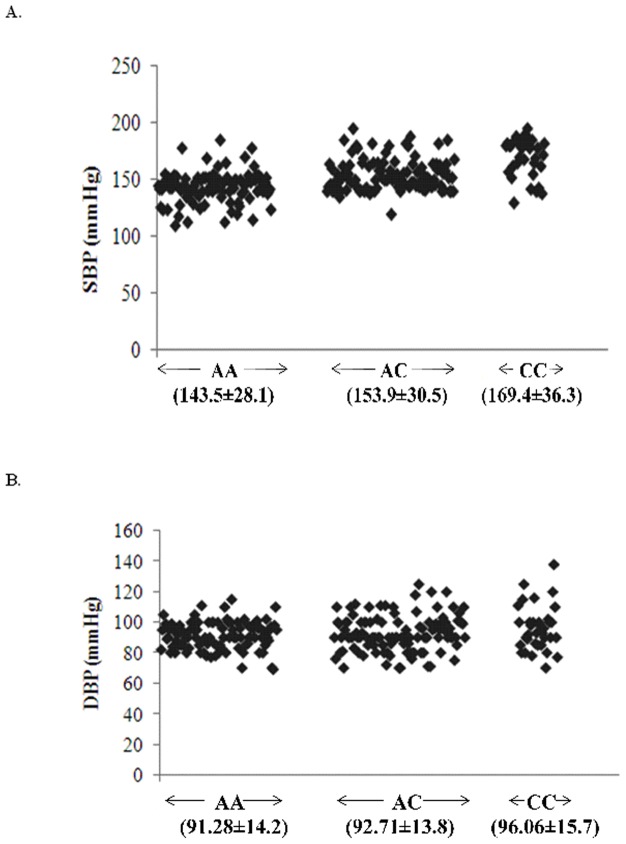
Intergenotypic (A1166C variant of the Angiotensin II type 1 receptor gene) variations in systolic and diastolic blood pressure in patients with essential hypertension. Figure 1A represents the scatter plot between Intergenotypic (A1166C variant of the Angiotensin II type 1 receptor gene) variations and systolic blood pressure. Figure 1B shows the scatter plot between Intergenotypic (A1166C variant of the Angiotensin II type 1 receptor gene) variations and diastolic blood pressure. Results were expressed as mean ± SD.

A statistically significant intergenotypic variation in the systolic blood pressure (SBP) in patients with AA, AC and CC genotypes was found to be 143.5±28.1 mmHg, 153.9±30.5 mmHg and 169.4±36.3 mmHg respectively. There was statistically significant results found between the genotypes and SBP when compared with AA Vs AC (p = 0.001), AA Vs CC (p = 0.0001) and AC Vs CC (p = 0.004), the p-values for AA Vs AC, AA Vs CC and AC Vs CC was found as 0.87, 0.10 and 0.44, respectively. The intergenotypic variation in the diastolic blood pressure (DBP) in patients with AA, AC and CC genotypes was found to be 91.28±14.2 mmHg, 92.71±13.8 mmHg and 96.06±15.7 mmHg respectively and was not statistically significant (p = 0.09) **(** **Figure 1** **)**.

### Angiotensin II type 1 receptor gene expression

**Figure 2:**
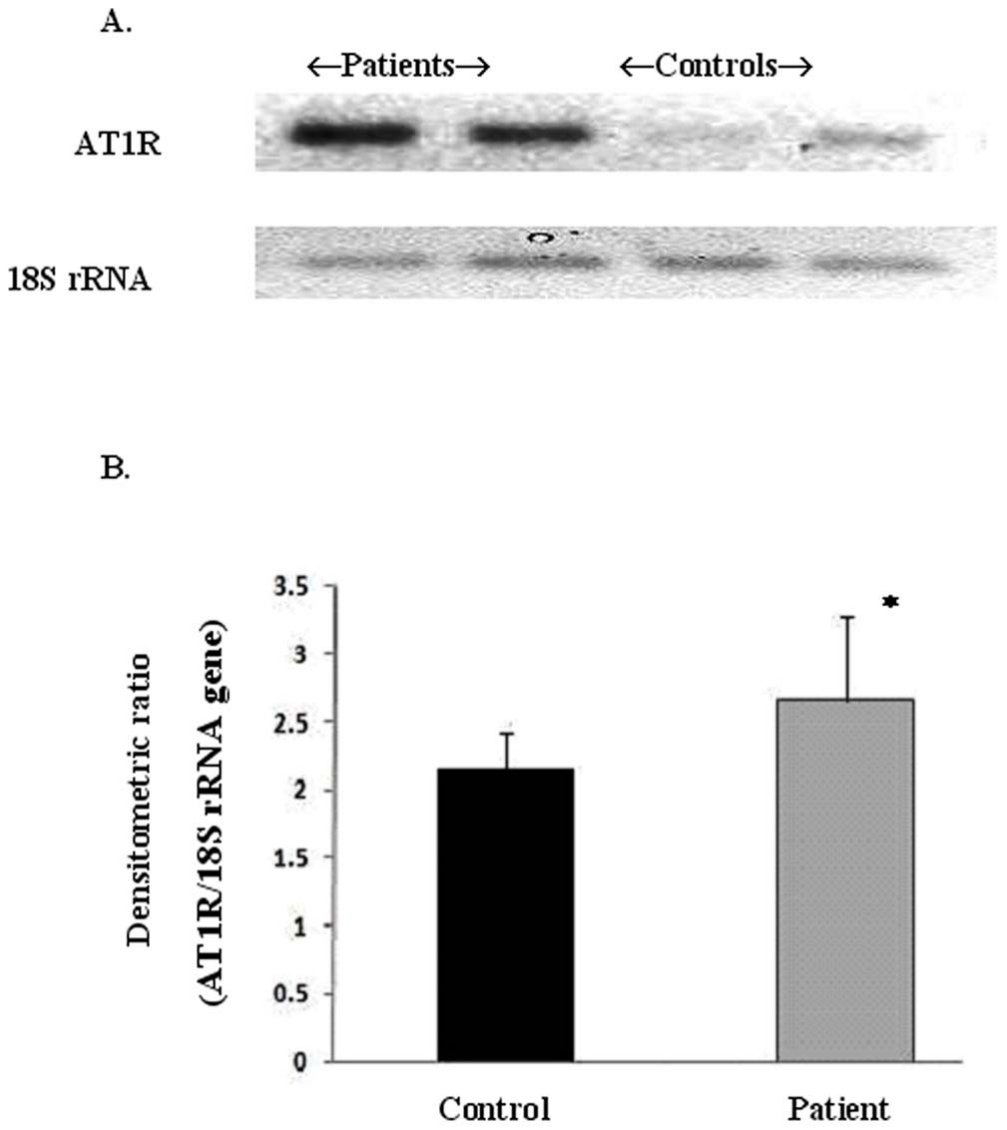
Semi-quantitative analysis of Angiotensin II type 1 receptor gene expression. Figure 2A represents the semi-quantitative analysis of AT1R gene. Figure 2B illustrates the relative AT1R gene expression in terms of integrated densitometric value (IDV) of patients (2.66±0.62) and normal healthy controls (2.15±0.28). Results were expressed as average densitometric ratio (AT1R to 18S rRNA gene) in patients and controls group ± SD. p<0.0001.

**Figure 3:**
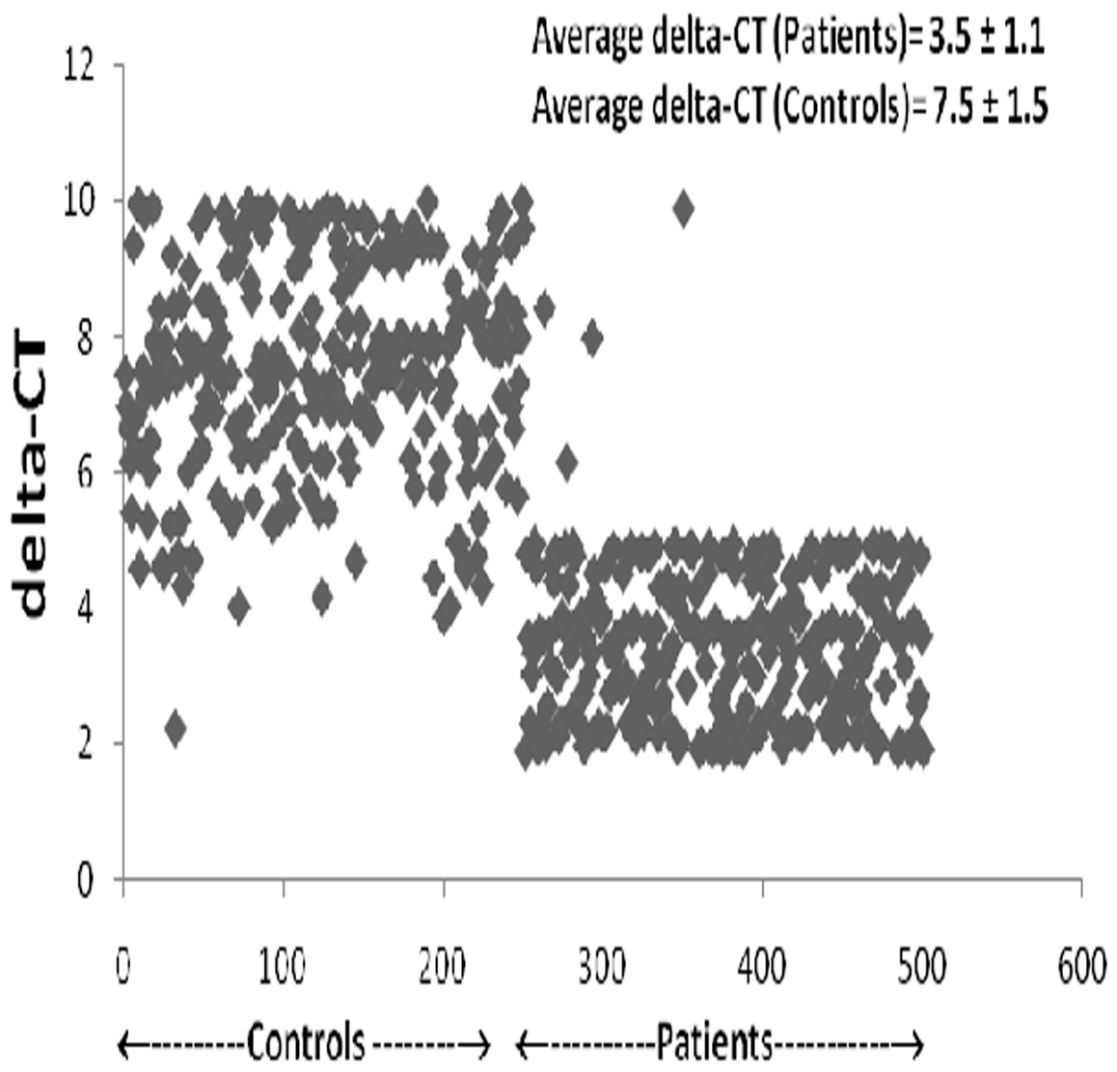
delta-CT values for study subjects. Figure 3 represents the scatter plot of delta-CT values for patients and controls group. The higher delta-CT value represents the lower expression of gene at mRNA level.

Figure 2 represents the semi-quantitative analysis of AT1R gene and housekeeping gene 18S rRNA. Semi-quantitative RT PCR indicated a small (1.2 times) but significant (p<0.0001) increase in the AT1R gene expression in patients (2.66±0.62) as compared to that of controls (2.15±0.28). Quantitative relative expression of AT1R gene, as calculated by delta-delta-CT method showed statistically significant difference between the average delta-CT value for patients and controls; 3.53±1.1 and 7.50±1.5 respectively (p = 0.0001) **(** **Table 3** **)**. The relative expression of mRNA for AT1R gene to 18S rRNA was increased by 15.6 fold in patients with essential hypertension as compared to that of controls. Figure 3 represents the scatter plot of delta-CT values for study subjects clearly indicating upregulation in the expression of AT1R gene in patient samples as compared to that of controls.

**Table 3:** Fold change expression and Quantitative PCR results of Angiotensin II type 1 receptor gene.

Subjects	Average deltaCT	delta-deltaCT	Fold difference
Patients (N = 250)	3.53±1.1*	−3.9	15.6
Controls (N = 250)	7.50±1.5*	0	1

CT, Threshold cycle.

*Data are means ± SD, *p = 0.0001. 18S rRNA gene expression of the same samples was used for calculations.

### Intergenotypic (A1166C variant of the Angiotensin II type 1 receptor gene) variations in gene expression (delta-CT values) in study subjects

**Figure 4:**
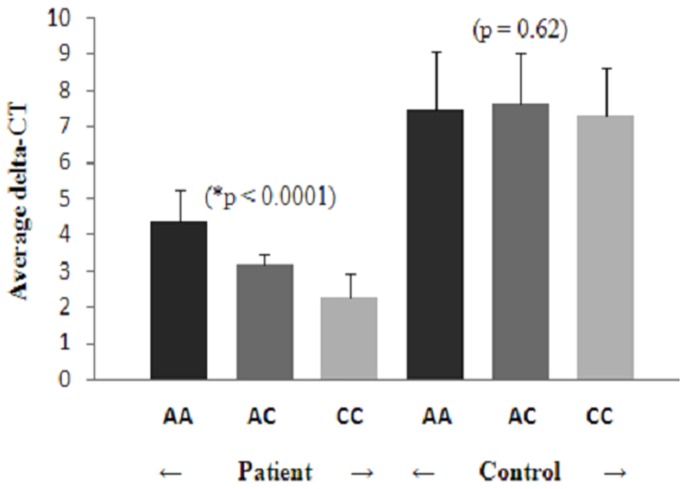
Intergenotypic (A1166C variant of the Angiotensin II type 1 receptor gene) variations in gene expression (delta-CT values) in study subjects. Figure 4 represents the Intergenotypic variations in gene expression (delta-CT values) in study subjects. Results were expressed as mean ± SD. *p<0.05 is considered to be significant.

The statistically significant intergenotypic variation in the delta-CT values was found in patients with AA, AC and CC genotypes, 4.36±0.90, 3.2±0.89 and 2.24±0.68 respectively (p = 0.0001). The intergenotypic variation in the delta-CT values in controls with AA, AC and CC genotypes was found to be 7.43±1.67, 7.66±1.37 and 7.29±1.34 respectively (p = 0.62) **(** **Table 4** **)**. As evident from figure 4, the CC genotype exhibits the maximum level of expression of AT1R gene as compared to AC and AA genotypes in patients with essential hypertension (p = 0.0001).

**Table 4:** Intergenotypic (A1166C variant of the Angiotensin II type 1 receptor gene) variations in gene expression (delta-CT values) in study subjects.

Subjects	Average delta-CT values in AT1R (A1166C) genotypes			p
	AA	AC	CC	
Patients (N = 250)	4.36±0.90	3.2±0.89	2.24±0.68	0.0001
Controls (N = 250)	7.43±1.67	7.66±1.37	7.29±1.34	0.62
**Comparison of Genotypes** *	**p**			
	**Patients**		**Controls**	
AA Vs AC	0.001		0.2	
AA Vs CC	0.001		0.1	
AA Vs AC+CC	0.001		0.09	

Patients and controls were compared with respect to genotypes with t-test of significance test at one degree of freedom adjusted for age and sex. p<0.05 is considered to be significant.

*analysis of variance (ANOVA) using Bonferroni’s method for multiple comparisons between genotype classes.

### Angiotensin II type 1 receptor protein expression

**Figure 5:**
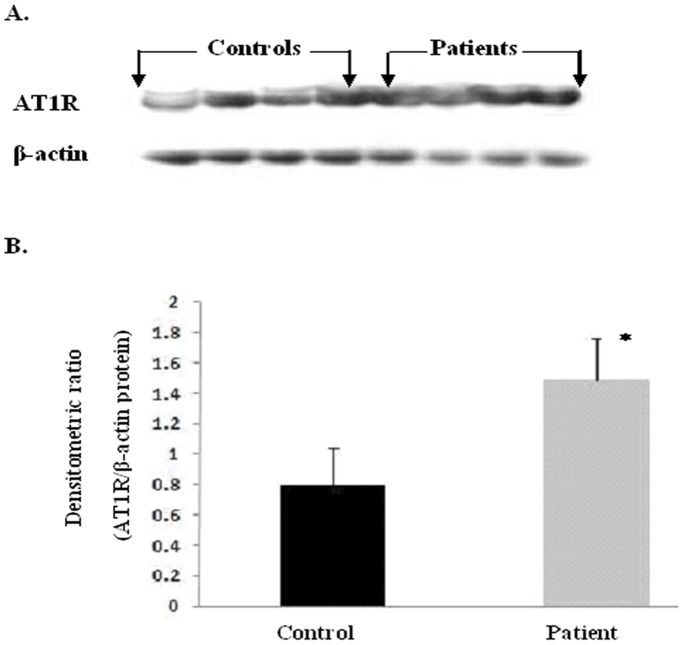
Western blot analysis of Angiotensin II type 1 receptor protein expression. Figure 5A represents the western blots of AT1R and β-actin protein in controls and patients respectively. Figure 5B shows the relative AT1R protein expression in terms of integrated densitometric value (IDV) of patients (1.49±0.27) and normal healthy controls (0.80±0.24). Results were expressed as average densitometric ratio (AT1R to β-actin protein) in patients and controls group ± SD. p = 0.0001.

Figure 5 shows a representative western blot of plasma AT1R protein of patients with essential hypertension and controls. Plasma AT1R protein expression levels were significantly different between the patient and control groups. The Integrated densitometric value (IDV) for AT1R protein was found to be 1.9 times higher in patients (1.49±0.27) as compared to the controls (0.80±0.24) (p = 0.0001). In contrast to mRNA levels, we did not observe a significant difference in protein levels of AT1R with respect to genotypes (AA, AC, and CC) in both the study subjects.

## Discussion

Gene polymorphism in AT1R with transversion of an adenine (A) to cytosine (C) at 1166 [23]–[24] is associated with cardiovascular diseases such as, coronary artery disease [42], myocardial infarction [43] and essential hypertension [24], [26]–[31]. To our knowledge, the present study is first of its kind, to determine the polymorphism, gene and protein expression of angiotensin II type 1 receptor in Northern Indian population. Study performed in the South Indian Tamilian population suggested that AT1R A1166C gene polymorphism is not associated with essential hypertension [35]. These findings are in contrast to other studies wherein A allele [44]–[46] and C allele [28], [47]–[48] have been reported to be predisposing factors for essential hypertension. We have conducted a case-control study to investigate the association between AT1R gene polymorphism, expression of AT1R at transcript and protein level and essential hypertension in an adequately powered study in North Indian population of similar socio-economical-geographical background. Mean values of baseline parameters were comparable in cases (patients with essential hypertension) and controls (normal healthy volunteers), except at the mean systolic and diastolic blood pressure levels.

In our study, we found that AA genotype was most frequent as compared to AC and CC genotypes in the controls group, while amongst patients AC genotype was most frequent compared to AA and CC genotypes. On further analysis we found that in our study the frequency of C allele in patients was 0.37 which is approximately 4 times higher than the study reported in Chinese Han [47] and Iranian [28] population. The Odds ratio observed in our study is 2.4, indicating that the individuals with CC genotypes may be at substantially higher risk of developing essential hypertension than those with the other two genotypes at the locus in question. The calculated odds ratio in our population was 1.3 times lower than that reported for the Tunisian population [48]. Importantly, we also observed intergenotypic variations in the mean systolic blood pressure in patients which once again indicates that the subjects having CC genotypes are more prone to develop essential hypertension.

It has been suggested that the AT1R gene expression has the major role in the vascular pathology and atherogenesis in case of Male apolipoprotein E/AT1AR double knockout mice [49]. In hypertensive animal studies, approximately 4 fold increase in expression of AT1R gene was found in hypothalalamus and 1.68 fold in adrenal medulla [36]. The upregulated levels of AT1R gene expression were also reported in case of hypercholesterolemia [50], and are associated with the functional role in the vascular smooth muscle cells [51]. Earlier studies have also shown that increased systolic blood pressure can be reversed by expression of AT1R antisense in adult spontaneously hypertensive rats [41]. Analysis of the expression of the AT1R, in the present study also showed a significant difference in the average delta-CT value of AT1R gene expression between patients and controls. We found a 15.6 fold up-regulation of AT1R gene expression in patients with essential hypertension in comparison to normal controls. We also observed a significant increase (p = 0.0001) in the expression of AT1R gene at protein level in patients with essential hypertension as compared to controls. The AT1R protein levels were increased up to 1.9 times in patients compared to control groups which are well supported by the studies indicating the role of AT1R protein expression in the regulation of blood pressure [39]–[40].

Importantly, the expression of AT1R gene was higher in patients with CC genotypes as compared to AC and AA. However, this trend was not observed in the control group. This further strengthens the fact that increased expression of AT1R is associated with essential hypertension. Our results are in contrast to those observed by Ceolotto et al. (2011) wherein authors did not observe any significant difference in the expression of AT1R at mRNA level in patients with CC genotypes compared to AA genotype. However, an increased protein expression was observed (70%) in patients with CC genotype [39]. The authors described this increase in AT1R expression to genotype specific decrease in expression of miR 155. Modulation/dysregulation of miR-155 were also reported in aorta of spontaneously hypertensive rats than in age – matched Wistar rats [52].

Essential hypertension being a polygenic disorder, difference in expression/mutations in other genes may be responsible for the observed difference in the expression of AT1R transcripts in Northern Indian population as compared to those reported by Ceolotto et al. [39]. As individuals with CC genotypes were relatively less than AC and AA genotypes in both the study subjects, our observations can at best be considered exploratory. It will also be interesting to check whether the expression of AT1R alters in patients after treatment. Further studies are needed to explore whether increased AT1R gene expression is an independent risk factor and can it serve as prognostic marker for essential hypertension. We envisage that studies in this direction may lead to better insight into the role of increased expression of AT1R in essential hypertension.

To conclude, our study shows a significant association of A1166C polymorphism in the angiotensin II Type 1 receptor gene with essential hypertension which is a major risk factor for cardiovascular diseases and strokes. We also observed an increased expression of AT1R gene in patients with C allele of A1166C polymorphism in Northern Indian population.

## Supporting Information

Table S1 Distribution of end-digit preference for systolic and diastolic blood pressure.
(DOC)
