# Supplementary material for: Association of Angiotensin II Type 1 Receptor (A1166C) Gene Polymorphism and Its Increased Expression in Essential Hypertension: A Case-Control Study
Source: PLoS One. 2014 Jul 3;9(7):e101502. doi: 10.1371/journal.pone.0101502 (PMC4081645; doi:10.1371/journal.pone.0101502)
Supplement: Table S1 — Distribution of end-digit preference for systolic and diastolic blood pressure. (DOC) [file pone.0101502.s001.doc]

**Table S1. Distribution of end-digit preference for systolic and diastolic blood pressure**

| Terminal digit | Subjects for SBP readings (N=500) | Percentage of subjects for SBP readings | Subjects for DBP readings (N=500) | Percentage of subjects for DBP readings |
| --- | --- | --- | --- | --- |
| 0 | 113 | 22.6 | 119 | 23.8 |
| 2 | 77 | 15.4 | 71 | 14.2 |
| 4 | 107 | 21.4 | 128 | 25.6 |
| 6 | 117 | 23.4 | 76 | 15.2 |
| 8 | 86 | 17.2 | 106 | 21.2 |

SBP, Systolic blood pressure; DBP, Diastolic blood pressure.
